# Thermal Change Affects Flexural and Thermal Properties of Fused Deposition Modeling Poly(Lactic Acid) and Compression Molding Poly(Methyl Methacrylate)

**DOI:** 10.1055/s-0042-1743148

**Published:** 2022-03-13

**Authors:** Taksid Charasseangpaisarn, Chairat Wiwatwarrapan, Viritpon Srimaneepong

**Affiliations:** 1 Dental Biomaterials Science, Graduate School, Chulalongkorn University, Bangkok, Thailand; 2 College of Dental Medicine, Rangsit University, Pathum Thani, Thailand; 3 Chula Unisearch, Chulalongkorn University, Bangkok, Thailand; 4 Department of Prosthodontics, Faculty of Dentistry, Chulalongkorn University, Bangkok, Thailand

**Keywords:** denture base, flexural modulus, flexural strength, fuse deposition modeling, temperature

## Abstract

**Objective** Polylactic acid (PLA) is one of the most widely used materials in three-dimensional (3D) printing technology due to its multiple advantages such as biocompatibility and biodegradable. However, there is still a lack of study on 3D printing PLA for use as a denture base material. The goal of this study was to compare 3D printing PLA to traditional poly(methyl methacrylate) (PMMA) as a denture basis.

**Materials and Methods** The PMMA (M) and PLA (L) specimens were fabricated by compression molding, and fuse deposition modeling technique, respectively. Each specimen group was divided into three different temperature groups of 25°C (25), 37°C (37), and 55°C (55). The glass transition temperature (T _g_ ) of raw materials and specimen was investigated using differential scanning calorimetry. The heat deflection temperature (HDT) of each material was also observed.

**Statistical Analysis** The data of flexural strength and flexural modulus were analyzed with two-way analysis of variance, and Tukey honestly significant difference. The T _g_ and HDT data, on the other hand, were descriptively analyzed.

**Results** The results showed that PLA had lower flexural strength than PMMA in all temperature conditions, while the PMMA 25°C (M25) and PMMA 37°C (M37) obtained the highest mean values. PLA 25°C (L25) and PLA 37°C (L37) had significant higher flexural modulus than the other groups. However, the flexural properties of L55 could not be observed, which may be explained by T _g_ and HDT of PLA.

**Conclusion** PLA only meets the flexural modulus requirement, although it was greater than flexural modulus of PMMA. On the other hand, PMMA can meet both good flexural strength and modulus requirement. However, increase in temperature could reduce flexural strength and flexural modulus of PMMA and PLA.

## Introduction

Increasing popularity of additive manufacturing, three-dimensional (3D) printing techniques, has also demonstrates good dimensional stability. 1 Fused deposition modeling (FDM) is one of the most popular additive manufacturing processes to fabricate a cost-effective thermoplastic work piece. 2 Chen et al compared the dimensional precision of the FDM and handmade fabrications of individual trays, and they found that 3D FDM had higher accuracy. 3 FDM-fabricated polylactic acid (PLA) is mostly used for prototypes or models due to simplicity of fabrication and low cost. 4 Apart from PLA, other polymeric materials that are widely manufactured by FDM include acrylonitrile-butadiene styrene, polyethylene terephthalate, thermoplastic polyurethane, and polyether ether ketone (PEEK).

PLA is a bio-based and biodegradable polymeric material that could be fabricated with many methods such as injection molding, compression molding, and 3D printing. In dentistry, PLA and related copolymers are available in the form of dental models, suturing materials, surgical barrier membrane, and fixation screw. 5 PLA has high flexural strength and flexural modulus according to a previous study. 6 The study by Deng et al showed that complete denture fabricated by FDM-PLA had high accuracy and adaptation to the cast. 2 Nevertheless, PLA has a low glass transition temperature (T _g_ ), which was reported to be less than 60°C, which is within the range of an intraoral temperature when eating or drinking hot food or beverages. 7 Even though PLA has many benefits, the investigation of PLA as a denture base material and the effect of temperature on the material have not been well studied.

Poly(methyl methacrylate) (PMMA) has been the most used polymeric material for denture bases. PMMA is known for its high flexural strength and modulus, as well as low water sorption and solubility. 8 The conventional heat-polymerized PMMA denture base resin can be fabricated either by compression molding, or injection molding techniques. 9 Until now, the residual monomer of PMMA still remains causing allergic reactions and toxicity to the oral tissues. 10 11

Thus, the objectives of this study were to investigate the thermal properties, flexural properties, and failure characteristics of PLA compared with PMMA at the different testing temperatures.

## Materials and Methods

### Sample Preparation

PMMA and PLA were prepared into bar-shaped specimens (size 63 × 10 × 3.3 mm) via different fabrication methods. Heat-polymerized PMMA specimens (Meliodent, Kulzer GmbH, Hanau, Germany) were made using compression molding technique and complete polymerization in water bath at 73.9°C for 9 hours following the manufacturer's instruction. The 3D printed PLA specimens (eSUN PLA, Shenzhen Esun Industrial Co., Shenzhen, China) were prepared by FDM method (Zortrax M200, Zortax S.A., Olsztyn, Poland) with 100% rectilinear infill pattern, in XY part orientation. The operating conditions of FDM machine are shown in Table 1 .

**Table 1:** Fuse deposition modeling processing conditions

Characteristic	Value
Chamber type	Close
Nozzle diameter (mm)	0.3
Nozzle speed (mm/s)	50
Extruder temperature (°C)	210
Bed temperature (°C)	60
Layer height (µm)	90

The specimens were refined and polished to the size of 64.0 × 10.0 × 3.3 mm with a Nano 2000T polisher (Pace Technologies, Arizona, United States), and metallographic paper P500, P1000, and P1200, accordingly. Before the test, all specimens were immersed in 37°C water incubator for 48 ± 2 hours.

### Flexural Properties Testing

Specimens of both materials, PMMA (M) and PLA (L), were divided into three different temperature groups including 25°C (25), 37°C (37), and 55°C (55). The specimens were immersed in temperature-controlled water bath for 1 minute before starting the test. The test was performed using a universal testing machine (Shimadzu EZ-S, Shimadzu Corp., Kyoto, Japan) with load cell 500 N and at cross-head speed of 5.0 mm/min. The testing method followed ISO 20795–1. 12 Three types of failure characteristic are classified in this study, which are as follows:

- Fracture (F): The specimen was totally broken and separated into two pieces.
- Tear (T): The specimen was partially damaged but still intact.
- Deflection (D): The specimen was bent without breaking.

### Differential Scanning Calorimetry (DSC) Measurement

The thermal properties of PMMA powder, PLA filament, and test specimens were investigated by differential scanning calorimeter (DSC) using DSC 204 F1 Phoenix (NETZSCH-Gerätebau GmbH, Selb, Germany) under nitrogen gas (flow rate 30 mL/min). T _g_ , crystallization temperature (T _c_ ), and melting temperature (T _m_ ) were determined by placing 10 mg of specimen in aluminum pan, and heating from 0 to 230°C with a rate of 5°C/min. The heating cycle was heat–cool–heat to erase the thermal history of the specimens, and data were gathered from second heat cycle.

### Heat Deflection Temperature Measurement

The heat deflection temperature (HDT) was measured in according with ISO 75–1. 13 The five specimens of each group of PMMA and PLA were prepared with the size of 80.0 × 10.0 × 4.0 mm. The 0.455 MPa load was applied to the specimen in the water bath. The testing temperature was heated from 21°C with a heat rate of 2°C/min until the specimens were deflected as stated in ISO 75–1. The data were recorded and represented as average value of HDT.

### Statistical Analysis

The flexural strength and flexural modulus were collected and analyzed with SPSS version 22 (IBM SPSS Statistics, IBM Corp., New York, United States). The flexural strength and flexural modulus were distinctly analyzed with two-way analysis of variance (ANOVA), with 95% confidence level.

## Results

The flexural properties of all specimens in the L55 group could not be detected due to deflection when force was applied, hence this group was excluded from the study and data were labeled as “not applicable” (N/A). The statistical analyses of the remaining groups revealed the normal distribution of data. The results of two-way ANOVA showed that the types of material and temperatures differed statistically between groups ( *p* < 0.05), and the interaction between the two factors also statistically differed ( *p* < 0.05) in both flexural strength and flexural modulus. As a result, all groups were subjected to a one-way ANOVA and a post-hoc test with Tukey honestly significant difference, as shown in Table 2 .

**Table 2:** Mean and standard deviation of flexural properties and characteristic of failure

Types of material	Testing temperature (°C)	Code	Flexural strength (MPa)	Flexural modulus (MPa)	Characteristics of failure a
PMMA	25	M25	74.05 ^A^ (5.69)	2,418.22 ^b^ (143.24)	F
	37	M37	73.84 ^A^ (2.87)	2,514.57 ^b^ (115.94)	F
	55	M55	56.82 ^C^ (1.92)	2,051.50 ^c^ (59.85)	F
PLA	25	L25	64.97 ^B^ (3.14)	2,763.59 a (168.07)	T
	37	L37	47.83 ^D^ (1.73)	2,612.13 a,b (280.03)	T
	55	L55	N/A	N/A	D

Abbreviations: N/A, not applicable; PLA, polylactic acid; PMMA, poly(methyl methacrylate).

Note: Similar superscript letters indicate that there is no significant difference between groups. The upper case and lower case superscript letters indicate separate statistical analyses.

a Characteristics of failure: F = fracture, T = tear, and D = deflection.

The maximum flexural strength values were found in M25 and M37, with no statistically significant difference between groups ( *p* > 0.05), but significantly greater than the other groups ( *p* < 0.05). The L25, M55, and L37 also exhibited statistically significant difference ( *p* < 0.05) with L25 being the most significant, followed by M55 and L37, respectively. L25 had the highest flexural modulus when compared with the other groups ( *p* < 0.05), except L37 ( *p* > 0.05). The L37, M37, and M25 revealed no statistically significant difference between groups ( *p* > 0.05), while M55 showed the lowest flexural modulus ( *p* < 0.05).

**Fig. 1:**
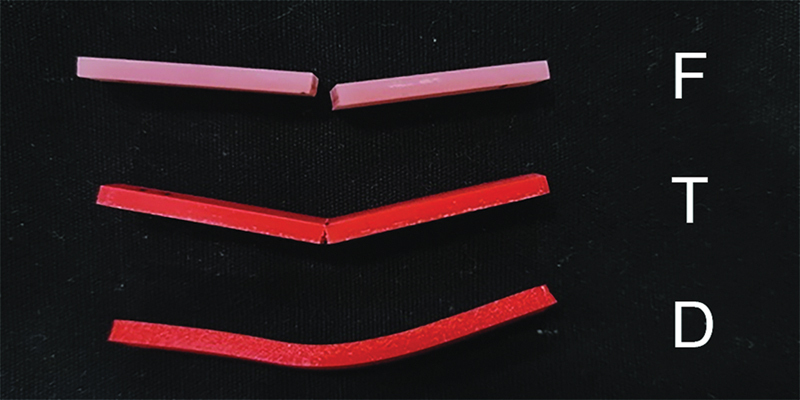
The sample of fracture (F), tear (T), and deflection (D) specimens.

When comparing the failure characteristic, all PMMA specimens underwent fracture after loading, whereas PLA specimens resulted in specimen deflection and eventually ruptured in group L25 and L37. However, L55 specimens displayed deflection of failure ( Fig. 1 ).

**Fig. 2:**
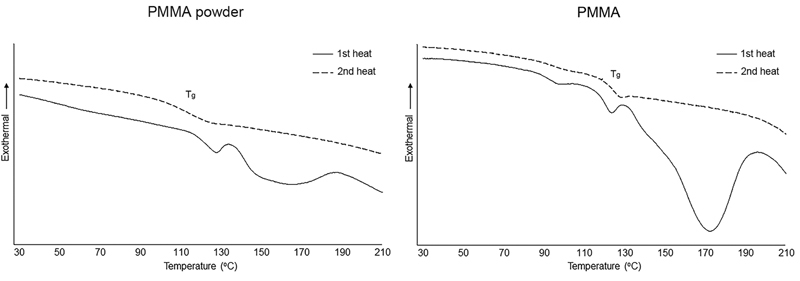
The differential scanning calorimeter thermograms of poly(methyl methacrylate) (PMMA) powder and PMMA specimen.

**Fig. 3:**
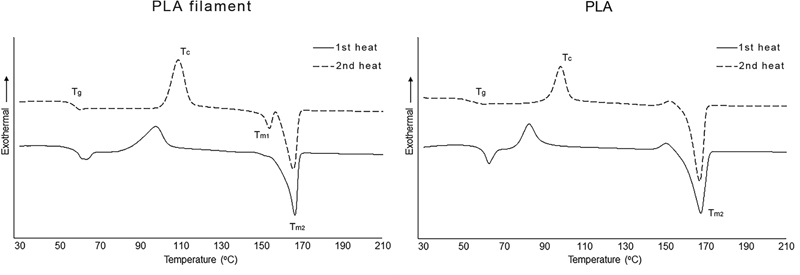
The differential scanning calorimeter thermograms of polylactic acid (PLA) filament and PLA specimen.

The HDT and thermal properties including T _g_ , T _c_ , and T _m_ of PMMA and PLA are shown in Table 3 . The DSC graph of PMMA and PLA including raw materials and specimens are presented in Fig. 2 and Fig. 3 accordingly.

**Table 3:** Thermal properties of PMMA and PLA specimens

Materials	HDT (°C)	Heat cycle	T _g_ (°C)	T _c_ (°C)	T _m1_ (°C)	T _m2_ (°C)
PMMA	–	1st heat	–	–	–	–
Powder	–	2nd heat	112.1	–	–	–
PMMA	104.20	1st heat	–	–	–	–
	(±0.37)	2nd heat	122.3	–	–	–
PLA	–	1st heat	57.3	97.5	–	166.5
Filament	–	2nd heat	55.0	108.7	153.8	165.6
PLA	55.02	1st heat	61.0	82.2	–	167.6
	(±0.28)	2nd heat	52.6	97.9	–	166.9

Abbreviations: HDT, heat deflection temperature; PLA, polylactic acid; PMMA, poly(methyl methacrylate).

## Discussion

This study was aimed to identify the effect of different temperatures that represent different situations on both mechanical and physical properties of PMMA and PLA materials. The temperature groups employed in this study were 25°C, 37°C, and 55°C, which represent the typical room temperature, human body temperature (according ISO 20795–1:2013), and the assumption of high intraoral temperature during the day, 14 respectively. The effect of temperature change on PMMA and PLA specimens was observed. When it came to PMMA, the M55 group had lower flexural strength and flexural modulus than the M37 and M25 groups. In case of PLA, L55 group specimens were distorted without breaking, therefore the measurement was not visible. The L37 group showed significantly lower flexural strength than L25 group, whereas the flexural modulus of L37 group was slightly lower than that of L25 with no statistically significant difference. The findings of the present study were consistent with those of Abdel-Wahab et al, who compared the testing temperatures of PMMA specimens from 20°C, 40°C, 60°C, and 80°C. The results showed that increasing temperature could gradually reduce the flexural strength, and led to a higher fracture strain. 15 However, PMMA showed higher flexural strength than PLA in this investigation. This was caused from the different fabricating methods. Fabrication by compression molding technique could produce the dense PMMA specimens. When testing at 25°C and 37°C, the flexural modulus of PMMA and PLA was not different. However, the flexural modulus of PMMA was significantly decreased when temperature raised to 55°C, unlike L55, which was deformed without breakage. This phenomenon could be explained by T _g_ . When the polymer is heated above its T _g_ , the polymer will act as rubber-like material and the polymer will be easily deflected when force is applied lowering the flexural properties. 16 The results could be explained by the fact that the T _g_ of polymer significantly influenced the flexural characteristics.

In this investigation, the T _g_ of PMMA powder as raw material and PMMA specimens was reported as 112.1°C and 122.3°C, respectively. Unlike the study of Durkan and Oyar, the T _g_ of PMMA was reported as 133°C. This could be attributed to the different powder to liquid ratio, polymerization method, and method of T _g_ measurement. 17 The T _g_ of PMMA specimen was higher than PMMA powder due to the higher degree of cross-linking from polymerization process. 18 The first heat scan of the DSC thermogram of PMMA ( Fig. 2 ) revealed an exothermic peak, whereas the second heat scan revealed no exothermic peak, while the transition zone was clearly observed. This phenomenon is corresponding to the previous studies by Ohyama and Imai, and Elshereksi et al. Their studies revealed that the exothermic peak was related to the benzoyl-peroxide and residual monomer, which remained after polymerization. 19 20 Thus, the exothermic peak of PMMA specimen was obviously observed comparing to PMMA powder due to the residual monomer from polymerization of methyl methacrylate monomer. However, the T _m_ of PMMA could not be detected due to the PMMA specimen being completely cross-linked thermoset material.

From the DSC thermogram of PLA ( Fig. 3 ), the T _g_ of PLA filament (raw material) and PLA specimen was 55.0°C and 52.6°C, accordingly. The T _g_ of PLA can be various, and it could be influenced by the crystallinity and molecular weight of PLA. 21 22 23 The T _g_ and T _c_ were observed in both first and second heat scans. The difference of T _g_ between PLA filament and PLA specimen may cause from the crystallinity that decreased after the PLA filament was melted to fabricate the specimen. This could be described by the peak area of T _c_ in PLA filament, which was greater than PLA specimen. The sharp peak of T _m_ was also observed in first and second heat scan temperatures between 165.6 and 167.6°C. The PLA filament displayed two peaks of T _m_ (T _m1_ and T _m2_ ) in second heat scan, which may be associated to the multiple crystalline phase formation (α and α') during crystallization mechanism of self-nucleation. 24 These results indicate that the PLA filament was semi-crystalline thermoplastic material. However, this was only observed in the PLA specimen. Although the T _g_ of PLA is less than PMMA, this was still higher than normal intraoral temperature.

The HDT is the temperature at which material begins to soften and deflect when loaded. The T _g_ differs from HDT in that T _g_ can be detected more than one values depending on the number of compositions in materials, but HDT could be shown as one value and can be different from the T _g_ . Thus, HDT is more appropriated to indicate the temperature of environment at which the materials can withstand the stress without deforming. The HDT of PMMA and PLA was explored in this study, and it showed that PMMA had higher HDT than PLA at high intraoral temperature, while the HDT of PLA was lower, which may cause deflection of material as found in L55 group. 14 However, in reality, the high intraoral temperature will be of short period and decrease immediately after swallowing the hot food or beverage. The testing condition was different because the controlled temperature was constant during testing for several minutes that allowed the distribution of more heat through the specimens.

The current study used PLA produced by the technology of FDM, which may have influence on a lower flexural strength. Comparing to the previous study of FDM-PLA, the flexural strength was closely to the result of L25 group at the testing temperature of 23°C. 25 However, some studies showed flexural strength of ∼95 MPa, which was higher than our result. This could be due to the fabrication method of injection molding of PLA. 26 This finding was supported by the study of Lay et al, who reported that the injection molding PLA had higher mechanical properties than FDM-PLA. 27 However, the comparison of flexural strength between injection molding and FDM is still limited.

By FDM process, the infill pattern, infill density, and part orientation are critical factors that influence the properties of the 3D printed materials. The study of Dave et al showed that 100% infill density, and rectilinear with XY orientation provided the highest mean tensile strength of printed part. 28 Hence, the parameters of 100% infill density and rectilinear infill pattern with XY printing direction were chosen to produce PLA in this study. Nugroho et al found that 500 µm layer thickness during printing gave significantly higher flexural strength of PLA than 100–400 µm layer thickness. 29 The larger layer thickness, on the other hand, results in a rougher surface on the PLA specimens, which requires more polishing and may impact dimension accuracy. However, Kuznetsov et al found that increasing the layer thickness could decrease the flexural strength of PLA specimens. 25 Therefore, the layer thickness of 90 μm was set up during printing of FDM-PLA specimens. Even though the layer thickness was set to the lowest thickness, the delamination or splitting of layer could commonly occur and may affect the flexural strength of printed part. 30

Additionally, following ISO 20795–1:2013, the specimens were assessed for pass or fail determination of flexural properties. 12 The ISO establishes the criteria for denture base material that the flexural strength should be more than 65 MPa, and flexural modulus should be more than 2,000 MPa for Type 1 (heat-activated) and Type 3 (thermoplastic) when tested in the 37°C water bath. Therefore, The M37 and L37 groups were chosen for this determination at a temperature of 37°C. The PMMA specimens met the flexural strength and flexural modulus requirements, while PLA only met the ISO flexural modulus criterion. Although FDM-PLA did not pass the ISO criteria of the flexural strength for denture base material, the flexural modulus was higher than PMMA. The limitations of this study were that this was a laboratory study, and the specimen was bar-shaped with 3.3 mm thickness, which is thicker from conventional denture base thickness. This could affect high mechanical strength. Further investigations should be performed on other fabrication techniques, such as injection molding or compression molding, which could yield superior flexural properties of the material. FDM-PLA has low thermal properties, but this could be improved by adding filler or modifier, which should be further investigated. For further study, other materials that could be fabricated by digital technology, especially PEEK, which represent good properties, color stability, and less abrasion, should be investigated comparing to PLA. 31 The recent study of Mirchandani et al have showed that the attachments of implant could be made of thermoplastic resin, therefore PLA may be an alternative material of attachment for implant. 32 This should be further studied. Additionally, use of FDM-PLA in the dental fields such individual tray, base plate, surgical template, or other applications that do not require high flexural strength, could be a promising alternative material of choice due to its biodegradable, environmentally nonpolluting property, and less health hazard than conventional PMMA.

## Conclusions

From the study, it could be concluded that:

1. The increased temperature from 25 to 37°C reduced the flexural strength of PLA but did not affect the flexural modulus. However, the increase in temperature from 25 to 37°C did not affect the flexural strength and flexural modulus of PMMA.
2. At the temperature of 55°C, the flexural properties of both PLA and PMMA were decreased. This should be considered for applications.
3. T _g_ and HDT of materials should be concerned when the application is performed at high temperature.
4. The FDM-PLA could possibly be an alternative denture base material, although heat-polymerized PMMA is still a standard of choice.
